# Orthognathic surgery and temporomandibular joint symptoms

**DOI:** 10.1186/s40902-015-0014-4

**Published:** 2015-05-28

**Authors:** Hwi-Dong Jung, Sang Yoon Kim, Hyung-Sik Park, Young-Soo Jung

**Affiliations:** 1 Department of Oral & Maxillofacial Surgery, Yonsei University College of Dentistry, Seoul, South Korea; 2 Private Practice Vienna VA; Former resident Harvard Oral & Maxillofacial Surgery, Boston, MA USA

## Abstract

The aim of this article is to review temporomandibular joint symptoms as well as the effects of orthognathic surgery(OGS) on temporomandibular joint(TMJ). The causes of temporomandibular joint disease(TMD) are multifactorial, and the symptoms of TMD manifest as a limited range of motion of mandible, pain in masticatory muscles and TMJ, Joint noise (clicking, popping, or crepitus), myofascial pain, and other functional limitations. Treatment must be started based on the proper diagnosis, and almost symptoms could be subsided by reversible options. Minimally invasive options and open arthroplasty are also available following reversible treatment when indicated.

TMD manifesting in a variety of symptoms, also can apply abnormal stress to mandibular condyles and affect its growth pattern of mandible. Thus, adaptive developmental changes on mandibular condyles and post-developmental degenerative changes of mandibular condyles can create alteration on facial skeleton and occlusion. The changes of facial skeleton in DFD patients following OGS have an impact on TMJ, masticatory musculature, and surrounding soft tissues, and the changes of TMJ symptoms. Maxillofacial surgeons must remind that any surgical procedures involving mandibular osteotomy can directly affect TMJ symptoms, thus pre-existing TMJ symptoms and diagnoses should be considered prior to treatment planning and OGS.

## Introduction

Dentofacial Deformity (DFD) is derived from many factors including genetic predisposition, environmental exposure, childhood facial trauma or infection, cyst or tumor, parafunctional habit causing developmental malocclusion, unilateral condylar hyperplasia, mandibular hypoplasia, prior surgical procedures, or temporomandibular joint disorder(TMD) [1]. Patients with dentofacial deformity (DFD) require an orthognathic surgery (OGS) for an improved facial profile and a correction of skeletal malocclusion and asymmetry. The motivating factors for patients undergoing OGS are to improve mastication, speech, and swallowing functions as well as facial esthetic and psychosocial factors [2]. The mandibular condyle is one of the anatomic structures that consist of TMJ, and the position of condyles in relation to temporal bone can be altered via various movement during OGS. Thus, OGS can affect both functional and esthetic components including mastication, promounciation, and TMJ functions.

Tempromandibular joint disorders (TMDs) include any clinical conditions associated with masticatory musculature, temporomandibular joint (TMJ), surrounding bony and soft tissue components, and any combinations of these structures. The symptoms of TMD manifest as a limited range of motion of mandible, pain in masticatory muscles and TMJ, Joint noise (clicking, popping, or crepitus), myofascial pain, and other functional limitations [3]. The positional changes of mandible, maxilla, or both jaws during OGS, can affect TMJ, masticatory musculature, its surrounding soft tissue, and TMD symptoms. Therefore, maxillofacial surgeons must carefully evaluate patients for presence of any TMJ symptoms preoperatively, and formulate treatment plans accordingly to prevent worsening of TMD symptoms. The purpose of this article is to review the publications on TMD as well as the effects of OGS on TMJ.

### Tempromandibular joint disorders

#### Causes & epidemiology

**Figure 1:**
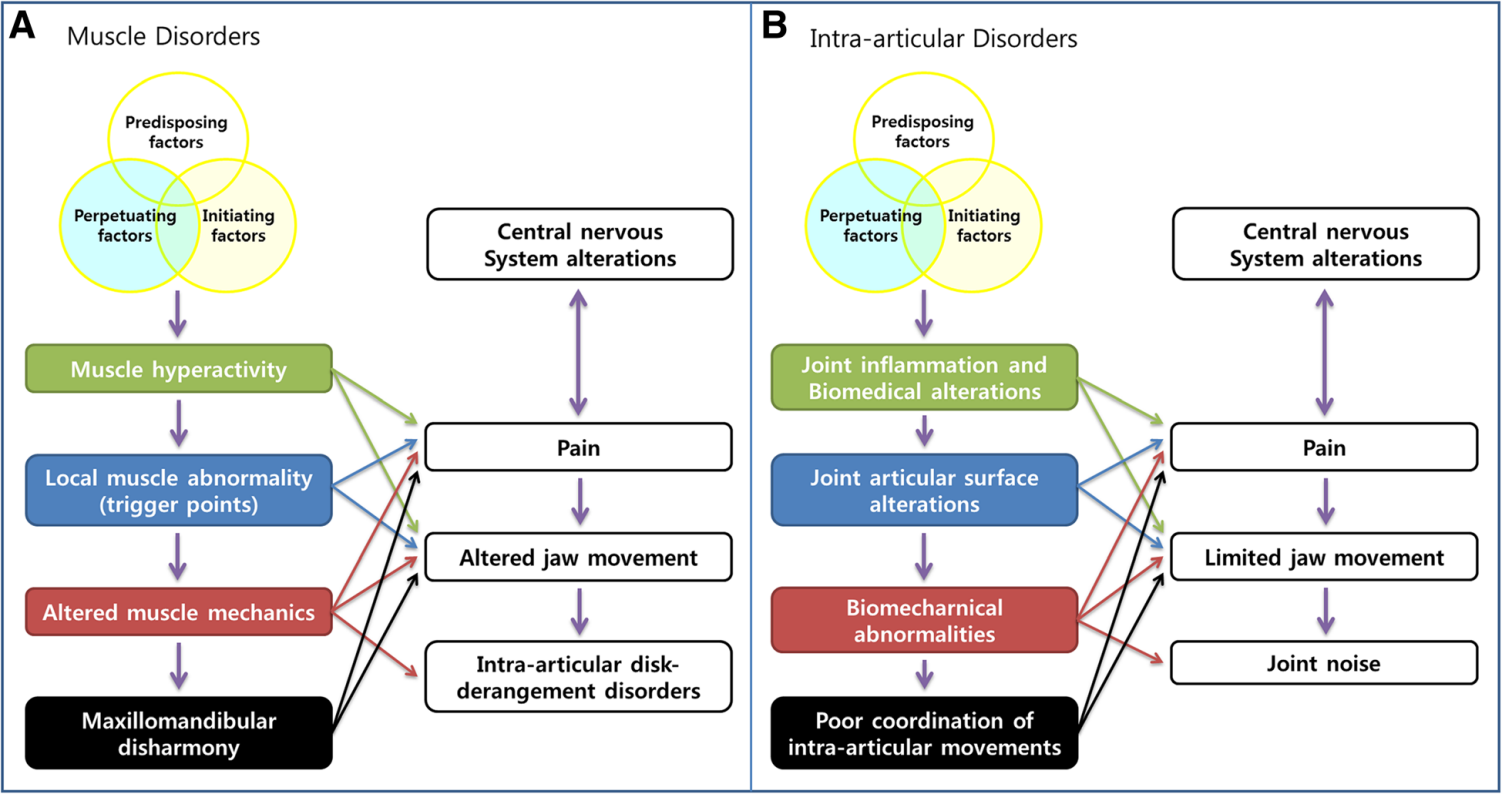
Pathogenesis of TMD. Adopted from Scrivani SJ, Keith DA, Kaban LB. Temporomandibular disorders. The New England journal of medicine 2008;359:2693–705 [4].

In 1930’s, JB Costen, an otolaryngologist, stated that TMD is a different disease process from otalgia, and is a condition derived from structural malalignment between mandible and cranium which requires a treatment coordination with dentists. The etiology of TMD was initially focused on dental occlusion for the next 50 years. However, it is now known that the causes of TMD are multifactorial including parafunctional habits (eg, nocturnal bruxing, tooth clenching, lip or cheek biting), emotional distress, acute trauma to the jaw, trauma from hyperextension (eg, dental procedures, oral intubations for general anesthesia, yawning, hyperextension associated with cervical trauma), instability of maxillomandibular relationships, laxity of the joint, comorbidity of other rheumatic or musculoskeletal disorders, poor general health, and an unhealthy lifestyle [4,5] (Figure 1)

.

Approximately 6 to 12% of population experience TMD symptoms, however only 5% of population manifests symptoms that require treatment [6]. TMD symptoms have a predilection for woman and age group between 20 to 40's [7]. Some study suggested an elevation of estrogen level in female patients [4] and hormonal influences as one of the causes of TMD [8-10], however this hypothesis has not been substantiated.

### Examination

Obtaining a detailed history from patients using TMD symptoms questionnaire is important prior to physical examination. During the initial consultation, chief complaints and history of present illness including TMD related symptom location, onset of occurrence, condition and character, alleviating or aggravating factors, and timing must be reviewed. Then, a focused physical examination is performed to identify the causes of symptoms and diagnosis. The range of motion of mandible is measured at active and passive maximum interincisal distance as well as at the onset of pain. When TMD symptoms are present, the location and onset of the pain are further investigated. The examination for muscles of mastication involves palpation of each muscle group and observation for any pain, spasms, or fasciculation. TMJ palpation is useful for identifying intracapsular pain, joint noise, and translation. Also TMJ loading test using tongue blade biting can be applied for evaluate intracapsular pain [11] (Table 1)

**Table 1:** **Physical examination directed toward mandibular dysfunction**

**Examination**	**Observations**
Inspection	Facial asymmetry, swelling, and masseter and temporal muscle hypertrophy Opening pattern (corrected and uncorrected deviations, uncoordinated movements, limitations)
Assessment of range of mandibular movement	Maximum opening with comfort, with pain, and with clinician assistance Maximum lateral and protrusive movements
Palpation examination	Masticatory muscles Temporomandibular joints Neck muscles and accessory muscles of the jaw Parotid and submandibular areas Lymph nodes
Provocation tests	Static pain test (mandibular resistance against pressure) Pain in the joints or muscles with tooth clenching Reproduction of symptoms with chewing (wax, sugarless gum)
Intraoral examination	Signs of parafunction (cheek or lip biting, accentuated linea alba, scalloped tongue borders, occlusal wear, tooth mobility, generalized sensitivity to percussion, thermal testing, multiple fractures of enamel, restorations)

From De Rossi S, Stern I, Sollecito TP. Disorders of the masticatory muscles. Dental clinics of North America 2013;57:449–64; and Data from references [4,6,111-115].

.

**Figure 2:**
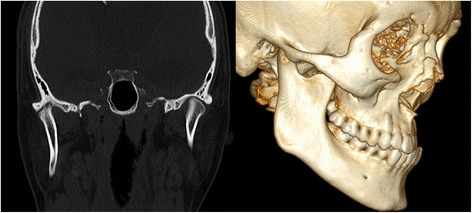
CT scan images of TMJ. Patients with history of trauma shows ankylosed TMJ on CT scan image, and 3D reconstruction demonstrates the overall shape of TMJ.

**Figure 3:**
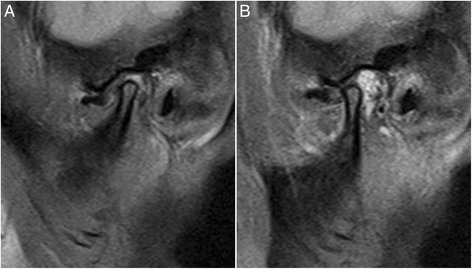
MRI Sagittal view showing disc displacement without reduction; **A**. Closed mouth; **B**. Open mouth.

Panoramic radiograph is a good screening tool for mandibular condyles and corresponding glenoid fossa relationship. For more detailed anatomic structure evaluation, multi-slice computed tomography (CT) or cone-beam computed tomography (CBCT) can be used. CT scans including CBCT is an excellent radiographic modality to evaluate mandibular condyle morphology, anatomic position, cortical erosion, presences of cyst or tumor, and ankylosis (Figure 2). The gold standard imaging modality for the disc and soft tissue surrounding TMJ is magnetic resonance image (MRI) [12], and the changes in disc position and location, morphology, and degenerative changes can be confirmed (Figure 3). However, MRI alone is not sufficient to formulate treatment plan, and other clinical findings are incorporated to make correct diagnoses and comprehensive treatment plans. MRI is not routinely performed on patients with DFD, thus clinical presentation, signs and symptoms, and standard radiographic images such as panoramic radiograph are used to make a correct diagnosis and implement further corresponding treatment modality [13] (Table 2)

**Table 2:** **AAOP diagnostic classification of TMDs**

**Diagnostic Category**	**Diagnoses**
Cranial bones (including the mandible)	Congenital and developmental disorders: aplasia, hypoplasia, hyperplasia, dysplasia (eg, first and second branchial arch anomalies, hemifacial microsomia, Pierre Robin syndrome, Treacher Collins syndrome, condylar hyperplasia, prognathism, fibrous dysplasia) Acquired disorders (neoplasia, fracture)
TMJ disorders	Deviation in form Disc displacement (with reduction; without reduction) Dislocation Inflammatory conditions (synovitis, capsulitis) Arthritides (osteoarthritis, osteoarthrosis, polyarthritides) Ankylosis (fibrous, bony) Neoplasia
Masticatory muscle disorders	Myofascial pain Myositis spasm Protective splinting Contracture

Adapted from Leeuw Rd, Klasser GD, American Academy of Orofacial P. Orofacial pain : guidelines for assessment, diagnosis, and management. 5th edition. Chicago: Quintessence Publishing; 2013.

.

### Treatment of Temporomandibular joint disorders

Once a correct diagnosis is made from detailed clinical data, initial treatment must be started with reversible options including patient education, medications, physical therapy, and occlusal splint therapy. Minimally invasive options (eg, trigger point injections, Botox injections, arthrocentesis, or arthroscopy) are available for TMJ pain and dysfunction, and also open arthroplasty can be performed as later options when indicated (Table 3)

**Table 3:** **Treatment of TMD symptoms in patients with dentofacial deformity**

**Reversible treatment of TMD**	**Irreversible treatment of TMD**
**Patient Education**	Trigger Point Injections
**Medications:**	Botox
Nonsteroidal anti-inflammatory drugs (anti-RA meds)	Arthrocentesis
Muscle relaxants	Arthroscopy
Antidepressants	Open Arthroplasty
**Physical Therapy**	
ROM exercised	
Passive stretching	
Spray and stretch	
Ultrasound	
Transcutaneous electrical nerve stimulation	
**Occlusal Splint Therapy**	

From Nale JC. Orthognathic Surgery and the Temporomandibular Joint Patient. Oral and maxillofacial surgery clinics of North America 2014;26:551–64 [116].

.

**Figure 4:**
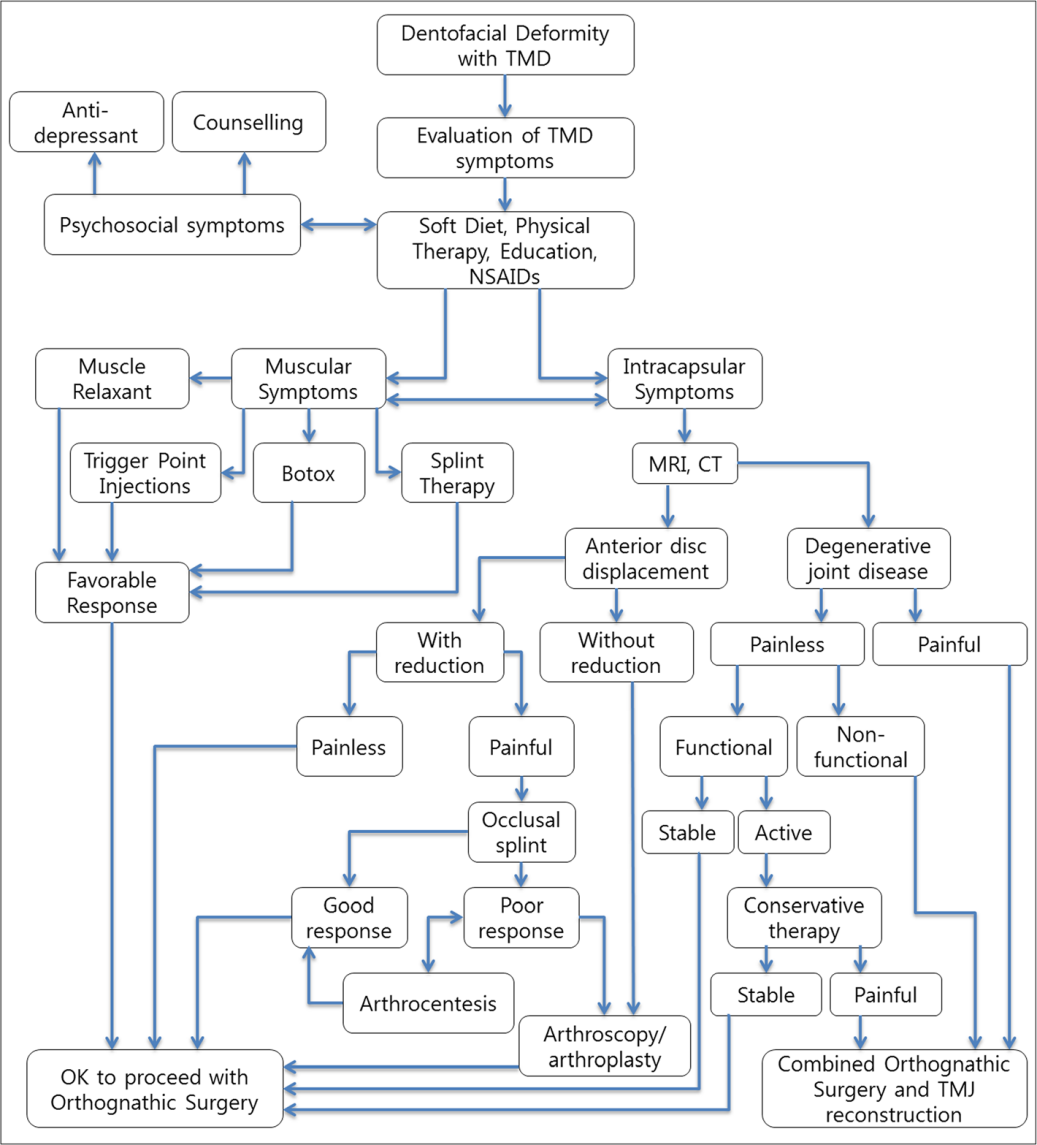
Flow diagram and treatment algorithm for patients with dentofacial deformity and TMD symptoms. Adopted from Nale, J.C. Orthognathic Surgery and the Temporomandibular Joint Patient. Oral and maxillofacial surgery clinics of North America 2014;26:551–64 [116].

Most TMD symptoms (approximately 85-90%) are treated with noninvasive, nonsurgical, and reversible interventions [13-15]. Patients with intra-articular disorder who has been refractory to nonsurgical treatment over 3 to 6 months with persistent pain and limited function would require a consideration for surgical interventions (Figure 4)

.

### Influences of temporomandibular joint disorder to dentofacial deformities

Adaptive developmental changes on mandibular condyles [16] and post-developmental degenerative changes of mandibular condyles can create alteration on facial skeleton and occlusion [17,18]. Also, trauma or developmental deformity causing the changes in morphology and occlusion, can alter biomechanics of TMJ, consequently develop into TMJ internal derangement (TMD ID) [19].

Skeletal class II malocclusion in children has higher propensity for TMD symptoms [20]. Also, skeletal class II malocclusion, longer posterior facial height, and hyperdivergent profile tend to show increased severity of TMJ ID [21]. In a retrospective study including children younger than 14, the presence of TMD affects a normal development of facial bones, and can result in mandibular asymmetry [18]. And Legrell PE and Isberg A also demonstrated a development of mandibular asymmetry from the condyle-disc complex disorder after surgically altering unilateral TMJ disc in animal study [22]. Thus, TMD manifesting in a variety of symptoms, also can apply abnormal stress to mandibular condyles and affect its growth pattern of mandible [23].

Patients with bilateral TMJ ID tend to have a short ramus, clockwise rotation of mandible, and retrognathic mandible [24-27], while patients with unilateral TMJ ID present lateral displacement of mandible and deviated occlusal and mandibular plane [19]. The severity of TMJ ID is also associated with amount of displacement of Antegonion and Menton [28,29], and the growth pattern can be altered in maxilla as well as mandible [30]. Unilateral TMJ ID displays a deviation of mention to the affected side [28,29,31]. And reverse examination study also demonstrated positive correlations between short ramus and condyle as well as deviated mention being associated with TMJ disc displacement and derangement [32,33]. Thus, the severity of TMD and disc displacement can lead to mandibular hypoplasia or facial asymmetry [18]. Also, degenerative changes and resorption of manduibular condyle beyond growth completion can lead to changes in skeletal shape [34].

### Influence of orthognathic surgery to temporomandibular joint

Many DFD patients desire to improve stomatognathic function and esthetics, as well as TMJ symptoms [35]. However, current literatures on the relationship between OGS and TMJ complications are still debatable [36]. Some authors claim that TMJ dysfunction can be improved after OGS, yet others claim deleterious effects on TMJ can occur after OGS [37,38].

Routine OGS procedure involves surgical movement of upper jaw via LeFort I osteotomy and lower jaw via ramus osteotomy. LeFort I osteotomy is not associated with direct trauma to TMJ or masticatory musculature, thus there are only minimal effects on TMJ dysfunction or mandibular movement [39]. Therefore, This review article focused on mandibular surgical modalities which directly affect the mandibular range of motion, mastication, and TMJ symptom changes.

### Sagittal split ramus osteotomy (SSRO)

SSRO is well known and very commonly used surgical technique worldwide for repositioning mandibular dental arch in both directions by advancement and setback movement of mandibular body [40]. SSRO provide a broad medullary contact between the bony segments that ensures stable healing capability. Internal fixation of bony segments eliminates or reduces the duration of intermaxillary fixation (IMF), plus a predictable immediate postoperative occlusion is achievable. The risk of neurovascular bundle injury is higher compared to intraoral vertical ramus osteotomy (IVRO) [41], and the risk of unfavorable fracture during the split between the bony segments was reported at 0.9% [42]. The risk of complications is reduced when experienced surgeons perform the procedure. Reproducing the original condylar position is difficult, and too much pressure can be placed against the articular disc or unfavorable condylar position can be created during SSRO. These conditions can potentially result in joint noise or pain, and can worsen any pre-existing TMD symptoms [43-47].

In association with mandibular setback surgery using SSRO, Ueki et al. [48] reported TMJ symptom relief in 66.7% of patients after SSRO, and Hu J et al. [49] reported symptom improvement in 40% of patients, yet a development of new TMJ symptom in 8% after SSRO. Kerstens et al. [50] reported 66% improvement of TMJ symptoms and 11.5% aggravation of symptoms while White and Dolwick [51] showed 89.1% improvement, 2.7% no changes, and 8.1% aggravation in TMJ symptoms. Although small degree of postoperative posterior or lateral displacement of condyle can be made following SSRO in class III patients, however these minor changes do not create significant changes in TMJ disc position or postoperative pain [35,52-54].

TMJ remodeling is divided into functional and dysfunctional remodeling. Dysfunctional remodeling has a significant alteration of the joint or occlusion and can cause reduction of condylar-ramus height, mandibular setback leading to class II malocclusion [55,56]. Dysfunctional remodeling is also known as condylar resorption which can be induced from systemic and local arthritis or trauma. Because a clear etiology is not present, it is categorized as idiopathic condylar resorption (ICR).

Maxillomandibular complex counterclockwise rotation via LeFort I osteotomy and SSRO can increase the mechanical loading of TMJ, and can lead to postoperative relapse [57]. Patients with systemic diseases such as rheumatoid arthritis, scleroderma, systemic lupus erythematosus, and other vascular collagenous diseases are known as high risk factors for condylar resorption [58-61]. Predisposing factors for ICR are the presence of TMJ dysfunction, young woman, high mandibular plane angle, and posteriorly inclined condylar neck [59,61-66].

A large amount of mandibular advancement via SSRO should be avoided to prevent condylar resoprtion occurring from the tension of stretched surrounding soft-tissue components [67-69]. Internal fixation with monocortical miniplates and screws (1.5-8.9%) showed more favorable response to condylar resoprtion than using bicortical screws (2–50.3%) during SSRO [70]. This is likely due to a torques being created on condyles from the proximal segment displacement during bicortical fixation [71]. The use of computer-aided design/computer-aided manufacturing-made condyle positioning jig has been suggested to minimize a significant condylar displacement or torque [72].

The postoperative relapse of open bite from condylar resorption usually occurs between 6 months to 3 year, thus a regular follow up is important to intervene early in the process [73]. Anti-inflammatory medication, tumor necrosis factor inhibitor, or matrix metalloproteinase inhibitor as pharmacotherapy [74], or an utilization of occlusal splints to reduce the joint loading [75] can help prevent resorption process. A total joint replacement option is also available if further active resorption process continues.

Zimmer et al. [76] reported that two-jaw surgery (maxillary advancement and mandibular setback surgery) had no influcence on mandibular mobility compared to a single-jaw surgery. Mandibular hypomobility is a common condition after mandibular advancement via SSRO especially with a prolonged IMF duration [77,78], degenerative changes during the periods of IMF [79], masticatory muscle unused atrophy, and decrease in muscle energy reserves due to immobilization [80,81]. Atrophy of human skeletal muscles and a decrease in strength and muscle energy reserves have also been associated with immobilization [80]. Aragon et al. recommended a sound postoperative rehabilitation program following orthognathic procedures to prevent hypomobility [82].

### Intraoral vertical ramus osteotomy (IVRO)

IVRO is one of mandibular osteotomy techniques commonly used for mandibular setback procedure [83-86]. A vertical osteotomy is made posterior to lingula, and proximal segment is placed lateral to distal segment without internal rigid fixation. It is relatively simple procedure and surgical time is much reduced compared to SSRO. Also, the risk of inferior alveolar nerve damage and neurologic deficit is lower [87,88]. Less than 1 mm of posterior relapse can occur after mandibular setback via IVRO, but the risk of further relapse is low and overcorrection is not commonly indicated [89]. Increased in transverse facial width from laterally positioned proximal segment is less than 1% due to continuing remodeling process [90].

A drawback of IVRO is the requirement of IMF since internal rigid fixation is not performed, and some clinicians recommend more than 4 weeks of IMF postoperatively [91-94]. However, active physical therapy with less than 2 weeks of IMF demonstrated a stable occlusion and good bone healing [95], (Table 4) Some study reported just one-day of IMF followed by early jaw exercise being sufficient [96]. When initial bite is unstable, an active physical therapy with close follow-ups, and re-IMF protocol is used to obtain improved occlusion, and 88% of patients achieve a stable occlusion after IVRO within 10 days of active physical therapy and Maximum mouth opening (MMO) more than 30 mm [95].

**Table 4:** **Active Physical Therapy instruction form for patients**

**Instructions for active physical therapy (Yonsei Protocol)**	
It has been about 2 weeks after undergoing your jaw surgery. The purpose of this active physical therapy is to help your facial musculatures and jaws adapt into a new position from the surgery. Please follow the instructions in order to recover your original jaw movement and stable result.	
1.	Open your mouth as big as possible : Repeat 3 times
A.	During the opening, check the lower incisal midline and do not allow laterally
B.	deviated movement.
C.	Close your mouth and lower tooth must be positioned into the splint without gap. If lower teeth are not positioned into the splint, try to close gap by pushing the jaw with your hands.
2.	Move your lower jaw anteriorly : Repeat 3 times. From the original position, move your lower jaw anteriorly and move back to its original position. Check the midline of the lower teeth and do not allow laterally deviated movement.
3.	Move your lower jaw to the left side : Repeat 3 times.
4.	Move your lower jaw to the right side : Repeat 3 times.
5.	Above instruction is 1 cycle. Please follow the instruction in order.
6.	You have to repeat above physical therapy protocol for 1 hour.
7.	Then, you have to fix the lower jaw to upper jaw for 2 hours.
8.	During the physical therapy, training elastics must be kept in the instructed site.
9.	Please avoid relatively hard food and be careful not to break the splint.
The splint is removed after 1 to 2 weeks of physical therapy, depending on the progess. It is not easy, but please be patient until finishing the physical therapy. This physical therapy is continued about 1 month and this therapy makes stable functional results.	
Department of Oral & Maxillofacial Surgery Dental Hospital, Yonsei Medical Center	

Adopted from Jung HD, Jung YS, Park JH, Park HS. Recovery pattern of mandibular movement by active physical therapy after bilateral transoral vertical ramus osteotomy. J Oral Maxillofac Surg 2012;70:e431-7.

IVRO requires a wide dissection of lateral aspect of ramus and medial aspect of proximal segment for muscle detachment. Freed proximal segment initially moves anterio-inferiorly and reduces the pressures on articular disc by physiologic equilibrium position and better condyle-disc relation [97]. Anterio-inferiorly moved condyle eventually returns back to its original position over time postoperatively [98].

Improvement of joint sound, pain, and other TMJ symptoms after IVRO is likely due to resting of TMJ and surrounding musculatures during IMF period as well as the condylotomy effects from anterio-inferior movement of condyle [39,88,97-100]. IVRO was reported to have 50-100% improvement of TMJ symptoms [101-104], and Jung et al. [105] reported 70.8% ~ 94.3% improvement of joint sounds and 89.4% improvement of TMJ pain after IVRO. Ueki et al. [48] reported TMJ symptom improvement in class III patients 88% after IVRO and 66.7% after SSRO, but MRI study showed 50% of improvement of anteriorly displaced disc after IVRO and no improvement after SSRO.

Horizontal condylar axis tend to be medially rotated when TMJ disc displacement or degenerative joint disease is present, and some authors suggested that medially rotated condyle is the etiological factor for TMD [106,107]. From this point of view, lateral rotation of condyle after IVRO is very effective improving TMJ symptoms. Choi et al. [108] evaluated 200 patients' postoperative changes in proximal segment and condyles on the transverse plane after IVRO using submentovertex cephalogram. This study reported 15.05 (SD: 8.97)° of postoperative lateral rotation of condyles which slowly returned towards the original position, yet 4.53 (SD: 6.03)° of lateral rotation remained at 1 year. This study included only class III malocclusion patients with low TMD prevalence and some patients were without known TMD. The condyles remained in laterally rotated position in all patients including the ones without known prior TMDs. Thus, laterally rotated condyles from IVRO improving TMD cannot be concluded from this study.

Condylar sagging in lateral or anterio-inferior direction can occur after IVRO. Condylar sagging can be avoided with careful dissection during the ramus osteotomy and not violating condylar capsules. In fact, the changes in the intercondylar distance on transverse plane after IVRO is not significant [108]. Excessive interference between the segments can induce sagging, thus reduction of bony interference or using a modified osteotomy design should be considered [109].

**Figure 5:**
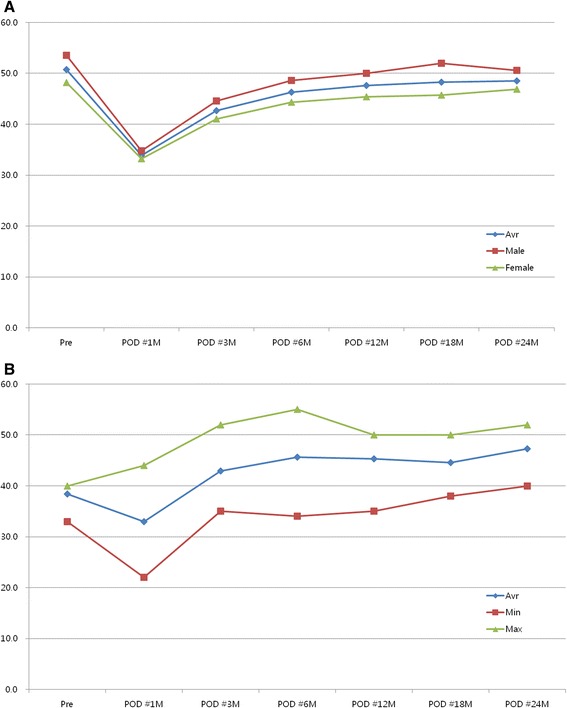
Recovery pattern following IVRO. **A.** Chronologic changes in the range of mandibular movement (maximal mouth opening). **B.** Chronologic changes in the range of mandibular movement (maximal mouth opening) in mandibular hypomobility patients. Abbreviations: Avr, average; POD, postoperative day. Adopted from Jung H et al. Recovery pattern of mandibular movement by active physical therapy after bilateral transoral vertical ramus osteotomy. Journal of oral and maxillofacial surgery 2012;70:e431-7 [95].

In order to prevent post OGS mandibular hypomobility, implementation of a sound postoperative rehabilitation program is very important [82]. The incidence of mandibular hypomobility after IVRO is very low and recovery of MMO is known to be 90-98% of pre-operative opening. Aragon et al. [82] showed 90% of recovery in 13 patients, Storum and Bell [110] showed 98% of recovery on 24 patients, Boyd et al. [39] showed 98% of recovery in 9 patients, and Jung et al. [95] reported 91.3% of recovery in 187 patients within 6 month and 95.7% recovery at 24 month after the procedure (Figure 5A). Patients with MMO less than 40 mm showed 112.5 to 123.2% recovery after IVRO procedure (Figure 5B)

.

## Conclusion

The changes of facial skeleton in DFD patients after OGS have an impact on TMJ, masticatory musculature, and surrounding soft tissues. Patients with TMJ symptoms requires a thorough evaluation including history taking, focused physical examination, and imaging modalities such as CT or MRI as indicated in order to obtain correct diagnoses and treatments prior to OGS. These evaluations are performed pre, intra, and post-operatively to determine the status of TMJ condition and managed appropriately. The changes of TMJ symptoms after OGS are associated with multiple factors including masticatory and facial musculature, and improvement in disc-condyle relationship as well as psychological factor. Any surgical procedures involving mandibular osteotomy can directly affect TMD, thus, pre-existing TMJ symptoms and diagnoses should be considered prior to treatment planning and OGS.

## References

[1] Pirttiniemi PM (1994). Associations of mandibular and facial asymmetries–a review. Am J Orthod Dentofac Orthop.

[2] Buttke TM, Proffit WR (1999). Referring adult patients for orthodontic treatment. J Am Dent Assoc.

[3] Wadhwa S, Kapila S (2008). TMJ disorders: future innovations in diagnostics and therapeutics. J Dent Educ.

[4] Scrivani SJ, Keith DA, Kaban LB (2008). Temporomandibular disorders. N Engl J Med.

[5] De Rossi S, Greenberg MS, Liu F, Steinkeler A (2014). Temporomandibular disorders: evaluation and management. Med Clin North Am.

[6] Lipton JA, Ship JA, Larach RD (1993). Estimated prevalence and distribution of reported orofacial pain in the United States. J Am Dent Assoc.

[7] Manfredini D, Guarda Nardini L, Winocur E, Piccotti F, Ahlberg J, Lobbezoo F (2011). Research diagnostic criteria for temporomandibular disorders: a systematic review of axis I epidemiologic findings. Oral surgery, oral medicine, oral pathology, oral radiology and endodontology.

[8] Abubaker AO, Raslan WF, Sotereanos GC (1993). Estrogen and progesterone receptors in temporomandibular joint discs of symptomatic and asymptomatic persons: a preliminary study. J Oral Maxillofac Surg.

[9] Milam SB, Aufdemorte TB, Sheridan PJ, Triplett RG, Van Sickels JE, Holt GR (1987). Sexual dimorphism in the distribution of estrogen receptors in the temporomandibular joint complex of the baboon. Oral surgery, oral medicine, oral pathology.

[10] Aufdemorte TB, Van Sickels JE, Dolwick MF, Sheridan PJ, Holt GR, Aragon SB (1986). Estrogen receptors in the temporomandibular joint of the baboon (Papio cynocephalus): an autoradiographic study. Oral surgery, oral medicine, oral pathology.

[11] De Rossi S, Stern I, Sollecito TP (2013). Disorders of the masticatory muscles. Dent Clin N Am.

[12] Tasaki MM, Westesson PL (1993). Temporomandibular joint: diagnostic accuracy with sagittal and coronal MR imaging. Radiology.

[13] Leeuw R, Klasser GD, American Academy of Orofacial P (2013). Orofacial pain. guidelines for assessment, diagnosis, and management.

[14] McCain JP, Sanders B, Koslin MG, Quinn JH, Peters PB, Indresano AT (1992). Temporomandibular joint arthroscopy: a 6-year multicenter retrospective study of 4,831 joints. J Oral Maxillofac Surg.

[15] Israel HA, Part I (1999). The use of arthroscopic surgery for treatment of temporomandibular joint disorders. J Oral Maxillofac Surg.

[16] Shen G, Darendeliler MA (2005). The adaptive remodeling of condylar cartilage–-a transition from chondrogenesis to osteogenesis. J Dent Res.

[17] Mongini F (1983). Influence of function on temporomandibular joint remodeling and degenerative disease. Dent Clin N Am.

[18] Schellhas KP, Pollei SR, Wilkes CH (1993). Pediatric internal derangements of the temporomandibular joint: effect on facial development. Am J Orthod Dentofac Orthop.

[19] Inui M, Fushima K, Sato S (1999). Facial asymmetry in temporomandibular joint disorders. J Oral Rehabil.

[20] Riolo ML, Brandt D, TenHave TR (1987). Associations between occlusal characteristics and signs and symptoms of TMJ dysfunction in children and young adults. Am J Orthod Dentofac Orthop.

[21] Jung WS, Kim H, Jeon DM, Mah SJ, Ahn SJ (2013). Magnetic resonance imaging-verified temporomandibular joint disk displacement in relation to sagittal and vertical jaw deformities. Int J Oral Maxillofac Surg.

[22] Legrell PE, Isberg A (1999). Mandibular length and midline asymmetry after experimentally induced temporomandibular joint disk displacement in rabbits. Am J Orthod Dentofac Orthop.

[23] Hellsing G, Holmlund A (1985). Development of anterior disk displacement in the temporomandibular joint: an autopsy study. J Prosthet Dent.

[24] Ahn S, Kim T, Nahm D (2004). Cephalometric keys to internal derangement of temporomandibular joint in women with Class II malocclusions. Am J Orthod Dentofac Orthop.

[25] Bryndahl F, Eriksson L, Legrell PE, Isberg A (2006). Bilateral TMJ disk displacement induces mandibular retrognathia. J Dent Res.

[26] Byun E, Ahn S, Kim T (2005). Relationship between internal derangement of the temporomandibular joint and dentofacial morphology in women with anterior open bite. Am J Orthod Dentofac Orthop.

[27] Yang I, Moon B, Lee S, Ahn S (2012). Skeletal differences in patients with temporomandibular joint disc displacement according to sagittal jaw relationship. J Oral Maxillofac Surg.

[28] Nakagawa S, Sakabe J, Nakajima I, Akasaka M (2002). Relationship between functional disc position and mandibular displacement in adolescent females: posteroanterior cephalograms and magnetic resonance imaging retrospective study. J Oral Rehabil.

[29] Trpkova B, Major P, Nebbe B, Prasad N (2000). Craniofacial asymmetry and temporomandibular joint internal derangement in female adolescents: a posteroanterior cephalometric study. Angle orthod.

[30] Maglione HO, de Zavaleta LA, Laraudo J, Falisi G, Fernandez F (2013). Temporomandibular dysfunction: internal derangement associated with facial and/or mandibular asymmetry. Cranio. J craniomandibular & sleep practice.

[31] Ahn S, Lee S, Nahm D (2005). Relationship between temporomandibular joint internal derangement and facial asymmetry in women. Am J Orthod Dentofac Orthop.

[32] Choi H, Kim T, Ahn S, Lee S, Donatelli RE (2011). The relationship between temporomandibular joint disk displacement and mandibular asymmetry in skeletal Class III patients. Angle orthod.

[33] Tallents RH, Guay JA, Katzberg RW, Murphy W, Proskin H (1991). Angular and linear comparisons with unilateral mandibular asymmetry. J craniomandibular disorders.

[34] Schellhas KP, Piper MA, Omlie MR (1990). Facial skeleton remodeling due to temporomandibular joint degeneration: an imaging study of 100 patients. AJNR Am J Neuroradiol.

[35] Farella M, Michelotti A, Bocchino T, Cimino R, Laino A, Steenks MH (2007). Effects of orthognathic surgery for class III malocclusion on signs and symptoms of temporomandibular disorders and on pressure pain thresholds of the jaw muscles. Int J Oral Maxillofac Surg.

[36] Iannetti G, Fadda TM, Riccardi E, Mitro V, Filiaci F (2013). Our experience in complications of orthognathic surgery: a retrospective study on 3236 patients. Eur Rev Med Pharmacol Sci.

[37] Dujoncquoy JP, Ferri J, Raoul G, Kleinheinz J (2010). Temporomandibular joint dysfunction and orthognathic surgery: a retrospective study. Head Face Med.

[38] Bays RA, Bouloux GF (2003). Complications of orthognathic surgery. Oral Maxillofac Surg Clin North Am.

[39] Boyd SB, Karas ND, Sinn DP (1991). Recovery of mandibular mobility following orthognathic surgery. J Oral Maxillofac Surg.

[40] Trauner R, Obwegeser H (1957). The surgical correction of mandibular prognathism and retrognathia with consideration of genioplasty. I. Surgical procedures to correct mandibular prognathism and reshaping of the chin. Oral surgery, oral medicine, oral pathology.

[41] Bell WH (1992). Modern practice in orthognathic and reconstructive surgery.

[42] MacIntosh RB (1981). Experience with the sagittal osteotomy of the mandibular ramus: a 13-year review. J Maxillofac Surg.

[43] Hackney FL, Van Sickels JE, Nummikoski PV (1989). Condylar displacement and temporomandibular joint dysfunction following bilateral sagittal split osteotomy and rigid fixation. J Oral Maxillofac Surg.

[44] Ellis E, Hinton RJ (1991). Histologic examination of the temporomandibular joint after mandibular advancement with and without rigid fixation: an experimental investigation in adult Macaca mulatta. J Oral Maxillofac Surg.

[45] Buckley MJ, Tulloch JF, White RP, Tucker MR (1989). Complications of orthognathic surgery: a comparison between wire fixation and rigid internal fixation. Int J Adult Orthodon Orthognath Surg.

[46] O'Ryan F, Epker BN (1983). Surgical orthodontics and the temporomandibular joint. II. Mandibular advancement via modified sagittal split ramus osteotomies. Am J Orthod.

[47] Feinerman DM, Piecuch JF (1995). Long-term effects of orthognathic surgery on the temporomandibular joint: comparison of rigid and nonrigid fixation methods. Int J Oral Maxillofac Surg.

[48] Ueki K, Marukawa K, Nakagawa K, Yamamoto E (2002). Condylar and temporomandibular joint disc positions after mandibular osteotomy for prognathism. J Oral Maxillofac Surg.

[49] Hu J, Wang D, Zou S (2000). Effects of mandibular setback on the temporomandibular joint: a comparison of oblique and sagittal split ramus osteotomy. J Oral Maxillofac Surg.

[50] Kerstens HC, Tuinzing DB, van der Kwast WA (1989). Temporomandibular joint symptoms in orthognathic surgery. J Craniomaxillofac Surg.

[51] White CS, Dolwick MF (1992). Prevalence and variance of temporomandibular dysfunction in orthognathic surgery patients. Int J Adult Orthodon Orthognath Surg.

[52] Kim Y, Yun P, Ahn J, Kim J, Kim S (2009). Changes in the temporomandibular joint disc position after orthognathic surgery. Oral surgery, oral medicine, oral pathology, oral radiology and endodontology.

[53] Ueki K, Nakagawa K, Takatsuka S, Yamamoto E (2006). The change of stress distribution on the condyle after mandibular setback surgery. Eur J Orthod.

[54] Fang B, Shen GF, Yang C (2009). Changes in condylar and joint disc positions after bilateral sagittal split ramus osteotomy for correction of mandibular prognathism. Int J Oral Maxillofac Surg.

[55] Arnett GW, Milam SB, Gottesman L (1996). Progressive mandibular retrusion–idiopathic condylar resorption. Part I. Am J Orthod Dentofac Orthop.

[56] Arnett GW, Milam SB, Gottesman L (1996). Progressive mandibular retrusion-idiopathic condylar resorption. Part II. Am J Orthod Dentofac Orthop.

[57] Hoppenreijs TJ, Stoelinga PJ, Grace KL, Robben CM (1999). Long-term evaluation of patients with progressive condylar resorption following orthognathic surgery. Int J Oral Maxillofac Surg.

[58] Lanigan DT, Myall RW, West RA, McNeill RW (1979). Condylysis in a patient with a mixed collagen vascular disease. Oral surgery, oral medicine, oral pathology.

[59] Huang YL, Pogrel MA, Kaban LB (1997). Diagnosis and management of condylar resorption. J Oral Maxillofac Surg.

[60] Haers PE, Sailer HF (1995). Mandibular resorption due to systemic sclerosis. Case report of surgical correction of a secondary open bite deformity. Int J Oral Maxillofac Surg.

[61] Kerstens HC, Tuinzing DB, Golding RP, van der Kwast WA (1990). Condylar atrophy and osteoarthrosis after bimaxillary surgery. Oral surgery, oral medicine, oral pathology.

[62] Moore KE, Gooris PJ, Stoelinga PJ (1991). The contributing role of condylar resorption to skeletal relapse following mandibular advancement surgery: report of five cases. J Oral Maxillofac Surg.

[63] Merkx MA, Van Damme PA (1994). Condylar resorption after orthognathic surgery. Evaluation of treatment in 8 patients. J Craniomaxillofac Surg.

[64] Hoppenreijs TJ, Freihofer HP, Stoelinga PJ, Tuinzing DB, van't Hof MA (1998). Condylar remodelling and resorption after Le Fort I and bimaxillary osteotomies in patients with anterior open bite. A clinical and radiological study. Int J Oral Maxillofac Surg.

[65] Hwang SJ, Haers PE, Sailer HF (2000). The role of a posteriorly inclined condylar neck in condylar resorption after orthognathic surgery. J Craniomaxillofac Surg.

[66] Hwang SJ, Haers PE, Zimmermann A, Oechslin C, Seifert B, Sailer HF (2000). Surgical risk factors for condylar resorption after orthognathic surgery. Oral surgery, oral medicine, oral pathology, oral radiology and endodontology.

[67] Kobayashi T, Izumi N, Kojima T, Sakagami N, Saito I, Saito C (2012). Progressive condylar resorption after mandibular advancement. Br J Oral Maxillofac Surg.

[68] Kawamata A, Fujishita M, Nagahara K, Kanematu N, Niwa K-i, Langlais RP (1998). Three-dimensional computed tomography evaluation of postsurgical condylar displacement after mandibular osteotomy. Oral Surgery, Oral Medicine, Oral Pathology, Oral Radiology, and Endodontology.

[69] Scheerlinck JPO, Stoelinga PJW, Blijdorp PA, Brouns JJA, Nijs MLL (1994). Sagittal split advancement osteotomies stabilized with miniplates. A 2–5-year follow-up. Int J Oral Maxillofac Surg.

[70] Joss CU, Vassalli IM (2009). Stability after bilateral sagittal split osteotomy advancement surgery with rigid internal fixation: a systematic review. J Oral Maxillofac Surg.

[71] Arnett GW, Gunson MJ (2013). Risk factors in the initiation of condylar resorption. Semin Orthod.

[72] Kim H, Baek S, Kim T, Choi J (2014). Evaluation of three-dimensional position change of the condylar head after orthognathic surgery using computer-aided design/computer-aided manufacturing-made condyle positioning jig. J Craniofac Surg.

[73] Hoppenreijs TJM, Stoelinga PJW, Grace KL, Robben CMG (1999). Long-term evaluation of patients with progressive condylar resorption following orthognathic surgery. Int J Oral Maxillofac Surg.

[74] Gunson MJ, Arnett GW, Milam SB (2012). Pathophysiology and pharmacologic control of osseous mandibular condylar resorption. J Oral Maxillofac Surg.

[75] Handelman CS, Greene CS (2013). Progressive/idiopathic condylar resorption: an orthodontic perspective. Semin Orthod.

[76] Zimmer B, Schwestka R, Kubein MD (1992). Changes in mandibular mobility after different procedures of orthognathic surgery. Eur J Orthod.

[77] Ueki K, Marukawa K, Hashiba Y, Nakagawa K, Degerliyurt K, Yamamoto E (2008). Assessment of the relationship between the recovery of maximum mandibular opening and the maxillomandibular fixation period after orthognathic surgery. J Oral Maxillofac Surg.

[78] White RP, Peters PB, Costich ER, Page HL (1969). Evaluation of sagittal split-ramus osteotomy in 17 patients. J Oral Surg.

[79] Glineburg RW, Laskin DM, Blaustein DI (1982). The effects of immobilization on the primate temporomandibular joint: a histologic and histochemical study. J Oral Maxillofac Surg.

[80] Lindboe CF, Platou CS (1982). Disuse atrophy of human skeletal muscle. An enzyme histochemical study. Acta Neuropathol.

[81] MacDougall JD, Ward GR, Sale DG, Sutton JR (1977). Biochemical adaptation of human skeletal muscle to heavy resistance training and immobilization. J Appl Physiol Respir Environ Exerc Physiol.

[82] Aragon SB, Van Sickles JE, Dolwick MF, Flanary CM (1985). The effects of orthognathic surgery on mandibular range of motion. J Oral Maxillofac Surg.

[83] Caldwell JB, Letterman GS (1954). Vertical osteotomy in the mandibular raml for correction of prognathism. J oral surg.

[84] Akin RK, Walters PJ (1975). Experience with the intraoral vertical subcondylar osteotomy. J oral surg.

[85] Tornes K, Gilhuus Moe OT (1987). The surgical technique of vertical subcondylar osteotomy for correction of mandibular prognathism. A 10-year survey. Acta Odontol Scand.

[86] Fonseca RJ, Marciani RD, Turvey TA, Scully JR (2009). Oral and maxillofacial surgery. Vol. 3.

[87] Astrand P, Ridell A (1973). Positional changes of the mandible and the upper and lower anterior teeth after oblique sliding osteotomy of the mandibular rami. A roentgen-cephalometric study of 55 patients. Scand J Plast Reconstr Surg.

[88] Hall HD, McKenna SJ (1987). Further refinement and evaluation of intraoral vertical ramus osteotomy. J Oral Maxillofac Surg.

[89] Jung H, Jung Y, Kim SY, Kim DW, Park H (2013). Postoperative stability following bilateral intraoral vertical ramus osteotomy based on amount of setback. Br J Oral Maxillofac Surg.

[90] Jung Y, Kim SY, Park S, Choi Y, Park H (2010). Changes of transverse mandibular width after intraoral vertical ramus osteotomy. Oral surgery, oral medicine, oral pathology, oral radiology and endodontology.

[91] Greebe RB, Tuinzing DB (1982). Overcorrection and relapse after the intraoral vertical ramus osteotomy. A one-year postoperative review of thirty-five patients. Oral surgery, oral medicine, oral pathology.

[92] Ayoub AF, Millett DT, Hasan S (2000). Evaluation of skeletal stability following surgical correction of mandibular prognathism. Br J Oral Maxillofac Surg.

[93] Lai SS, Tseng Y, Huang IY, Yang Y, Shen Y, Chen C (2007). Skeletal changes after modified intraoral vertical ramus osteotomy for correction of mandibular prognathism. J Plast Reconstr Aesthet Surg.

[94] Chen C, Lee H, Yang C (2008). Intraoral vertical ramus osteotomy for correction of mandibular prognathism: long-term stability. Ann Plast Surg.

[95] Jung H, Jung Y, Park JH, Park H (2012). Recovery pattern of mandibular movement by active physical therapy after bilateral transoral vertical ramus osteotomy. J Oral Maxillofac Surg.

[96] Ohba S, Tasaki H, Tobita T (2013). Assessment of skeletal stability of intraoral vertical ramus osteotomy with one-day maxillary-mandibular fixation followed by early jaw exercise. J Craniomaxillofac Surg.

[97] Ueki K, Marukawa K, Shimada M (2007). Condylar and disc positions after intraoral vertical ramus osteotomy with and without a Le Fort I osteotomy. Int J Oral Maxillofac Surg.

[98] Bell WH, Yamaguchi Y (1991). Condyle position and mobility before and after intraoral vertical ramus osteotomies and neuromuscular rehabilitation. Int J Adult Orthodon Orthognath Surg.

[99] Astrand P, Ericson S (1974). Relation between fragments after oblique sliding osteotomy of the mandibular rami and its influence on postoperative conditions. Int J Oral Surg.

[100] Egyedi P, Houwing M, Juten E (1981). The oblique subcondylar osteotomy: report of results of 100 cases. J oral surg.

[101] Bell WH, Yamaguchi Y, Poor MR (1990). Treatment of temporomandibular joint dysfunction by intraoral vertical ramus osteotomy. Int J Adult Orthodon Orthognath Surg.

[102] Hall HD, Navarro EZ, Gibbs SJ (2000). Prospective study of modified condylotomy for treatment of nonreducing disk displacement. Oral surgery, oral medicine, oral pathology, oral radiology and endodontology.

[103] Hall HD, Navarro EZ, Gibbs SJ (2000). One- and three-year prospective outcome study of modified condylotomy for treatment of reducing disc displacement. J Oral Maxillofac Surg.

[104] Choi Y, Yun K, Kim S (2002). Long-term results of different condylotomy designs for the management of temporomandibular joint disorders. Oral surgery, oral medicine, oral pathology, oral radiology and endodontology.

[105] Jung H, Jung Y, Park H (2009). The chronologic prevalence of temporomandibular joint disorders associated with bilateral intraoral vertical ramus osteotomy. J Oral Maxillofac Surg.

[106] Westesson PL, Liedberg J (1987). Horizontal condylar angle in relation to internal derangement of the temporomandibular joint. Oral surgery, oral medicine, oral pathology.

[107] Fernández Sanromán J, Gómez González JM, del Hoyo JA (1998). Relationship between condylar position, dentofacial deformity and temporomandibular joint dysfunction: an MRI and CT prospective study. J Craniomaxillofac Surg.

[108] Choi YS, Jung H, Kim SY, Park H, Jung Y (2013). Remodelling pattern of the ramus on submentovertex cephalographs after intraoral vertical ramus osteotomy. Br J Oral Maxillofac Surg.

[109] Jung H, Kim SY, Park H, Jung Y (2014). Modification of intraoral vertical ramus osteotomy. Br J Oral Maxillofac Surg.

[110] Storum KA, Bell WH (1986). The effect of physical rehabilitation on mandibular function after ramus osteotomies. J Oral Maxillofac Surg.

[111] Schiffman EL, Fricton JR, Haley DP, Shapiro BL (1990). The prevalence and treatment needs of subjects with temporomandibular disorders. J Am Dent Assoc.

[112] Kurita K, Westesson PL, Yuasa H, Toyama M, Machida J, Ogi N (1998). Natural course of untreated symptomatic temporomandibular joint disc displacement without reduction. J Dent Res.

[113] Milam SB, Laskin DM, Greene CS, Hylander WL (2006). TMJ osteoarthritis. Temporomandibular disorders : an evidence-based approach to diagnosis and treatment.

[114] Rammelsberg P, LeResche L, Dworkin S, Mancl L (2003). Longitudinal outcome of temporomandibular disorders: a 5-year epidemiologic study of muscle disorders defined by research diagnostic criteria for temporomandibular disorders. J Orofac Pain.

[115] Clark GT, Seligman DA, Solberg WK, Pullinger AG (1989). Guidelines for the examination and diagnosis of temporomandibular disorders. J Craniomandib Disord.

[116] Nale JC (2014). Orthognathic surgery and the temporomandibular joint patient. Oral Maxillofac Surg Clin North Am.

